# Supplementary material for: Comprehensive Draft Genome Analyses of Three Rockfishes (Scorpaeniformes, Sebastiscus) via Genome Survey Sequencing
Source: Curr Issues Mol Biol. 2021 Nov 18;43(3):2048–58. doi: 10.3390/cimb43030141 (PMC8929126; doi:10.3390/cimb43030141)
Supplement: Supplementary file 1 [file cimb-43-00141-s001.zip › cimb-1411902-supplementary.pdf]

Table S1 Base composition of *Sebastiscus* mitochondrial genomes.

	S. albofasciatus					*S. tertius*					*S. marmoratus*				
	**A**	**T**	**G**	**C**	**A+T**	**A**	**T**	**G**	**C**	**A+T**	**A**	**T**	**G**	**C**	**A+T**
Whole	28.15	26.59	17.00	28.26	54.73	28.21	26.48	16.76	28.55	54.69	28.48	26.49	16.64	28.39	54.97
12S rRNA	29.81	19.87	22.62	27.70	49.68	29.67	19.96	22.70	27.67	49.63	30.02	19.77	22.41	27.80	49.79
16S rRNA	32.62	21.16	20.98	25.24	53.78	32.45	21.22	21.16	25.18	53.66	32.74	20.98	20.86	25.41	53.72
COI	24.95	30.37	17.86	26.82	55.32	24.63	30.37	18.12	26.89	55.00	25.02	30.63	17.79	26.56	55.64
COII	28.94	28.22	16.35	26.48	57.16	28.80	28.08	16.50	26.63	56.87	28.36	28.36	16.79	26.48	56.73
COIII	25.10	27.39	17.32	30.19	52.48	25.61	27.77	17.07	29.55	53.38	25.48	27.64	16.94	29.94	53.12
ATPase 6	26.94	29.72	13.32	30.01	56.66	26.94	29.72	13.62	29.72	56.66	27.09	29.14	13.03	30.75	56.22
ATPase 8	28.57	23.21	13`.69	34.52	51.79	29.17	23.21	13.10	34.52	52.38	29.17	22.02	13.10	35.71	51.19
ND1	24.82	27.90	15.49	31.79	52.72	24.72	27.28	15.28	32.72	52.00	24.82	27.59	15.49	32.10	52.41
ND2	27.25	26.20	12.62	33.94	53.44	27.34	26.48	12.43	33.75	53.82	26.77	26.77	13.10	33.37	53.54
ND3	22.06	32.66	15.76	29.51	54.73	21.78	32.66	15.76	29.80	54.44	21.78	32.95	16.05	29.23	54.73
ND4L	21.89	28.28	16.84	33.00	50.17	20.88	28.28	17.17	33.67	49.16	22.22	27.61	16.50	33.67	49.83
ND4	26.21	28.60	15.06	30.12	54.82	25.92	28.31	15.35	30.41	54.24	26.36	27.66	14.84	31.14	54.02
ND5	27.95	27.24	14.46	30.34	55.19	27.95	26.75	14.25	31.05	54.70	28.28	27.08	14.25	30.40	55.36
ND6	14.56	36.78	33.52	15.13	51.34	14.37	37.55	34.10	13.98	51.92	14.75	38.51	33.33	13.41	53.26
Cyt b	24.89	30.06	15.43	29.62	54.95	25.24	30.15	15.34	29.27	55.39	24.80	30.85	15.43	28.92	55.65
